# Utilizing DNA analysis to combat the world wide plague of present day slavery – trafficking in persons

**DOI:** 10.3325/cmj.2014.55.3

**Published:** 2014-02

**Authors:** Timothy Palmbach, Jeffrey Blom, Emily Hoynes, Dragan Primorac, Mario Gaboury

**Affiliations:** 1 University of New Haven, West Haven, CT, USA; 2 Global Sentry Group, Inc., El Paso, TX, USA; 3 Department of Pediatric Medicine, University of Split, School of Medicine, Split, Croatia; 4 University of Osijek, School of Medicine, Osijek, Croatia

## Abstract

A study was conducted to determine if modern forensic DNA typing methods can be properly employed throughout the world with a final goal of increasing arrests, prosecutions, and convictions of perpetrators of modern day trafficking in persons while concurrently reducing the burden of victim testimony in legal proceedings. Without interruption of investigations, collection of samples containing DNA was conducted in a variety of settings. Evidentiary samples were analyzed on the ANDE Rapid DNA system. Many of the collected swabs yielded informative short tandem repeat profiles with Rapid DNA technology.

## Trafficking in persons as a major societal issue

Through efforts of government agencies, dedicated non-profit organizations, and various forms of media the reality and horrors of trafficking in persons (TIP) is becoming common knowledge. The scope of the problem is daunting; to some extent footprints of slavery can be seen in every country to some extent. TIP is always associated with force, fraud, or coercion and generally takes one of two forms: sex trafficking and forced labor.

Reported numbers of individuals who are currently in bondage as a result of some form of modern slavery are as high as 29.8 million (1). This estimate already appears astounding, yet there are good indicators that even this astounding number may not fully capture the extent of this problem. Effective identification of victims of human trafficking remains a challenge. In fact, last year only 47 000 of the potential 27 million victims were adequately identified (2). One of the major objectives of the US State Department and United Nations Office on Drugs and Crime (UNODC) is to gather sufficient evidence to accurately identify and account for victims of present day slavery. DNA typing by short tandem repeat (STR) analysis is the most powerful and reliable tool available today in human identification and is accepted globally in criminal investigations and judiciary proceedings as a source of forensic evidence to support or refute an individual’s involvement in criminal activities. What is more, an individual’s DNA is inherited from their mother and father, and as such DNA typing can be used to genetically verify claimed familial relationships. The use of DNA typing and the development of supporting databases will offer reliable and effective forensic/genetic evidence to support TIP victim identification, family reunification, and prosecution of the TIP perpetrators (3).

There are several potential methods to help identify and account for these victims. One such method is STR technology, also known as “DNA typing.” Law enforcement agencies have developed databases of STR profiles from individuals and sample materials of interest. STR profiles have been employed primarily to match criminal suspects with evidentiary samples to investigate crime, exonerate the innocent, and provide evidence in judicial proceedings. The success of the approach has led to its expansion to a number of applications, including human trafficking.

An example of the power of DNA can be seen in the work of DNA-Prokids. DNA-Prokids is an organization that has successfully utilized DNA profiling to return missing or illegally adopted children back to their parents. The stated overall mission of DNA-Prokids is to utilize genetic identification of victims and their families to fight against human trafficking. Ultimately they desire to create and manage a worldwide database of victims (4). In addition to this utility, DNA profiling is a powerful tool that can be of great value to investigators and prosecutors involved in criminal cases of human trafficking.

## Current problems associated with trafficking of persons investigations

Investigative and prosecutorial initiatives involving TIP cases have relied heavily upon traditional law enforcement methods such as surveillance, use of informants, and interview and interrogation of victims and alleged perpetrators. These methods have proven to be valuable for investigators, and will continue to be of value even with the integration of newer methods such as DNA analysis. Several notorious locations where commercial sexual exploitation of children has occurred, such as those in Svay Pak, Cambodia have been greatly impacted through use of these traditional investigative means. However, the scope and magnitude of the problem will require far greater resources and enhanced technologies to even begin to keep up with this continually evolving criminal enterprise. As the sophistication of human trafficking grows, so must the investigative resources. Some of these resources should include tools available to the criminal justice community through forensic science, specifically forensic DNA analysis.

Achieving an acceptable level of success in deterring and stopping human trafficking will require policies and procedures to address a number of longstanding issues including:

1) Inability to accurately determine the number of current victims of TIP, and the number of victims who have passed in and out of trafficking for a variety of reasons.

2) Inability to positively identify victims due to inadequate missing persons databases.

3) Difficulty in tracking the movement of victims from one location to another both within and across national boundaries.

4) Difficulty in identifying individuals engaged in human trafficking and/or the sex trade.

5) Need for a reliable method to identify recruitment and transportation patterns; that is to identify key routes of trafficking victims and perpetrators.

6) Need for procedures to collect physical evidence that will be beneficial in the identification of individuals participating in the sex trafficking trade.

7) Lack of resources available to analyze physical evidence for prosecution or other governmental actions.

8) Lag time between acquiring biological evidence and obtaining STR profiles.

9) Need to leverage the identification of perpetrators involved in human trafficking to lead to their arrest and prosecution as well as raising public awareness and improving government programs for the prevention of TIP.

10) Need to minimize further trauma to victims as a result of prosecution and reducing the chances of acquittal from victim refusal to testify by utilizing DNA analysis and other investigative methods as key components of the prosecution.

Modern forensic DNA analysis can provide a means to address many issues associated with human trafficking and sexual exploitation, and potentially other forms of modern day slavery such as forced labor. Substantial advances in DNA analysis over the past two decades have provided forensic laboratories and the criminal justice community with powerful tools with which to analyze physical evidence. With the advent of low copy number (LCN) or touch DNA, mitochondrial DNA, and Y-STR methods, it is now common practice to obtain viable DNA profiles from very minute or mixture sources commonly encountered at crimes scenes. Many of these procedures are specifically designed to address samples associated with sexual assault evidence, the same type of evidence that can be obtained from sexually exploited victims and or the locations where these crimes occur. In addition, these methods can be used to identify suspects from objects they contacted such as cigarette butts or water bottles.

Further, with the advent of Rapid DNA technologies it is now possible to conduct forensic quality STR analysis outside the laboratory by nontechnical personnel. In addition, rapid DNA analysis can generate STR profiles in less than two hours in a field forward setting, a major benefit to ongoing investigations.

STR profiles generated from evidence can be placed into and searched against appropriate DNA databases. Further, under some circumstances, databases can be shared across jurisdictional or country boundaries, and used for a variety of identification and investigative purposes.

## Use of DNA evidence in sexual assault and child exploitation

For the past two decades, DNA typing has played an increasing role in the successful outcome of a variety of criminal matters including those involving sexual assault or related offenses. Early applications in domestic violence and sexual assault cases generated criticism and skepticism. But over time, the value of DNA evidence in criminal investigations and prosecutions has been recognized.

A study by Johnson et al attempted to evaluate the use of forensic science in investigating crimes of sexual violence. Only 31% of the cases they reviewed had evidence examined by a forensic science laboratory. They concluded that the presence of forensic evidence in a given case had a positive effect on the overall evidentiary strength of the case. Examination of forensic evidence increased the odds of a District Attorney filing charges in the case by over five times. Further, their study indicated that a strong case included traditional values such as victim cooperation and availability of witnesses, as well as the presence of biological evidence (5).

An additional study stated that while rape cases involving the analysis of evidence were 11 times more likely to result in a District Attorney’s choice to prosecute, in a majority of the cases the decision to prosecute occurred before laboratory analysis. This study also concluded that rape cases with forensic evidence had an overall 81.4% conviction rate while cases without forensic evidence had a 64.3% conviction rate (6). There was no indication as to the extent of the delay in obtaining DNA results. It would be interesting to determine the effect of Rapid DNA analysis through which viable DNA results could be obtained within a few days of the crime, generally well before an arrest occurs, and observe the impact made on conviction rates and decisions to prosecute.

Another study addressed how mock jurors in a case involving the alleged sexual assault of a 6-year-old child used DNA evidence in comparison to the victim’s testimony. Their study concluded, “The impact of DNA evidence on a child sexual assault trial is powerful but not absolute.” However, in both experiments conducted there was an indication that the jurors perceived DNA evidence as more important than the victim’s testimony (7).

There is no silver bullet in the successful investigation of crimes of violence including rape and sexual exploitation. Studies have shown the value of forensic evidence, including DNA evidence, but have cautioned that other factors will remain critical to an investigation and successful prosecution. TIP cases involving sexual exploitation are criminal matters that involve the same activities and thus have the same potential for retrieving physical evidence as the rape cases highlighted in the above noted studies, as well as other studies. Thus, there is every reason to expect that the use of DNA analysis will be of value in the investigation, arrest, prosecution, and conviction of those accused of TIP violations involving sexual exploitation. The extent and scope of that evidentiary value may vary from case to case, but this can also be said currently for “traditional” sexual assault cases.

Despite the similarities between “traditional” sexual assault cases and cases involving the sexual exploitation of TIP victims, there are differences in investigative strategies and methodologies. One such methodology is performing undercover work to obtain information from the victims and perpetrators themselves. This kind of work will require modification of current evidence collection and preservation protocols to be effective in the undercover arena and extended nature of human trafficking investigations. The essential purpose of the research is to determine if modern forensic DNA typing methods can be properly employed throughout the world, with a final goal of increasing arrests, prosecutions, and convictions of perpetrators of modern human trafficking while concurrently reducing the trauma of victims and the burden of victim testimony in legal proceedings.

## Methods for evidence collection in TIP cases

We used standard evidence collection and preservation supplies and methods, with modifications as required to address specific issues encountered in TIP cases while working in remote locations. These methods have recently been utilized in fieldwork associated with a country assessment of TIP issues in Costa Rica and Nepal.

### Reference DNA samples from TIP victims

**Figure 1:**
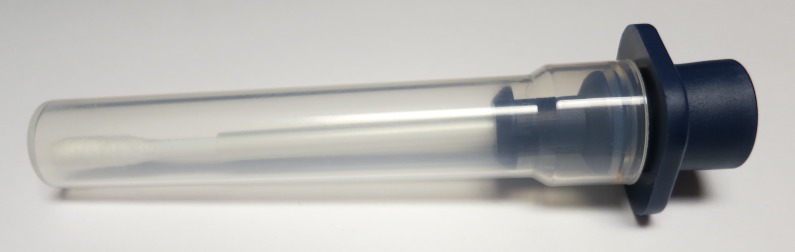
View of a Net Bio BioChipSet swab.

Collection of reference samples can be obtained from TIP victims through a simple buccal swab. Swabs were obtained from NetBio (Waltham, MA, USA). (Figure 1). These known samples can be collected by a variety of service personnel with a minimum amount of training who come in contact with the victims. Trained investigative personnel obtained samples during ongoing undercover investigative efforts. In some instances, trained undercover personnel took the buccal samples from victims with their consent. Other times, after having been informed of the process and having voluntarily consented to the sample collection, the victims stated they were more comfortable collecting the sample themselves, and were subsequently allowed to do their own buccal swab collection under the careful observation of the trained undercover personnel.

If necessary, where it is not practical to obtain reference swabs from a victim, personal objects can be processed. Cigarette butts, drinking glasses, or water bottles are good sources. Investigative personnel were able to determine that a particular item was in direct contact with the suspected TIP victim. Objects were surreptitiously swabbed in place and then left in the given establishment. Investigative personnel left with only the buccal swab. In addition to collection of a DNA sample, investigators obtained other supporting investigative information and captured many of the transactions on concealed surveillance equipment.

### Collection of DNA samples from objects within brothels, etc.

**Figure 2:**
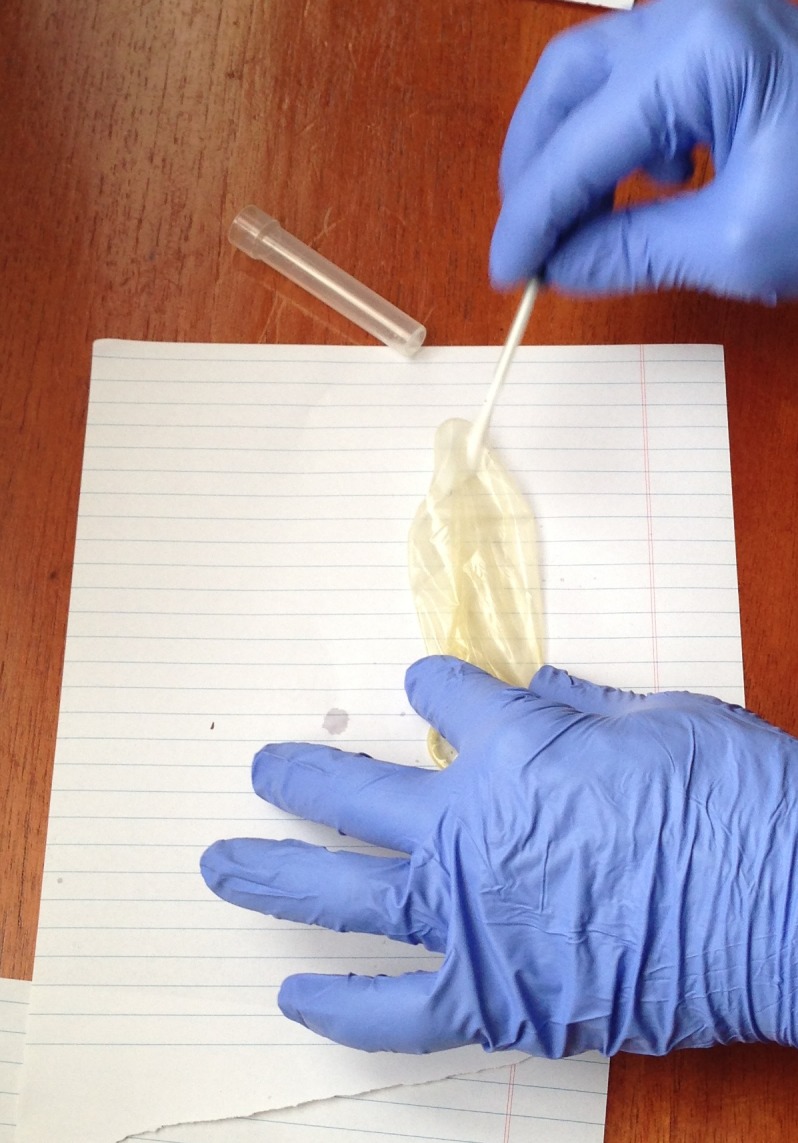
Swabs obtained from recovered condoms.

Biological samples suitable for DNA typing were collected from brothels or locations believed to house or transport victims. Samples included used condoms and tissues. In most cases, investigators swabbed the samples and transported the resulting swabs to the involved laboratories (Figure 2).

In hotels, bars, or other establishments known to be gathering spots for individuals engaged in commercial sexual exploitation, objects left in public spaces, such as cigarette butts or water bottles may be collected for subsequent DNA analysis.

After the initial swabs were obtained, obvious signs of excess moisture and subsequent bacterial growth were noted. If conditions in the field, and realities associated with undercover operations, did not allow for sufficient air-drying time after sample collection, procedures were modified to use a commercially available swab storage tube that contained a drying agent.

### DNA analysis

**Figure 3:**
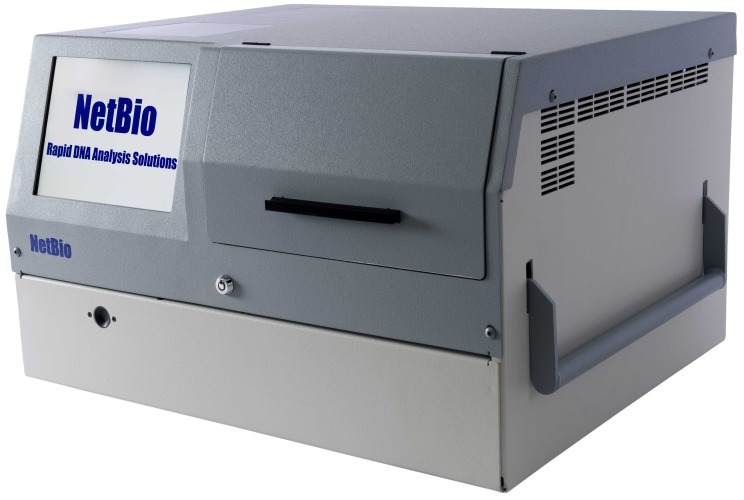
ANDE Rapid DNA Analysis System.

Rapid DNA Analysis was performed at NetBio using the field forward ANDE Rapid DNA Analysis System (NetBio, Waltham, MA, USA) (Figure 3) consisting of the automated ANDE instrument and Low Content DNA biochips. Note that none of the samples shown in this paper have been used in criminal proceedings. However, collection, preservation, and analysis of samples were conducted in a manner consistent with future use of similar samples in investigations and/or legal proceedings.

### Undercover collection of samples

Investigative personnel were able to enter establishments that were known to contain or were suspected of containing victims who were likely trafficked and being subjected to sexual exploitation. Locations included brothels, massage parlors, cabin restaurants, sport clubs, nightclubs and bars, and dance bars. Many of these locations had security personnel located at the entrance and subjected all patrons to some form of security check. These activities ranged from a physical pat down to electronic screening for weapons. Thus, investigators had to transport swabs and distilled water ampules in locations and methods that would not be detected by these search procedures. In all instances investigative personnel were able to enter and exit facilities without detection of the swabs and supplies. When it was necessary to process items for DNA inside an establishment such as swabbing an empty beer or water bottle, it had to be done in a manner to avoid detection and suspicion. Obtaining reference samples from TIP victims was successfully done after gaining the confidence of the victims without drawing attention from security personnel or managers of the TIP victims. For obvious reasons it is imperative that the collection of these types of samples in these environments be conducted by properly trained investigative personnel.

### Rapid DNA analysis

An example of Rapid DNA analyses is presented in Table 1. Investigative personnel obtained a total of 50 samples during their operations in Costa Rica and Nepal. Nineteen of those samples were from known buccal swabs from TIP victims, and these samples yielded a 95% success rate for obtaining full STR profiles and 5% success rate for partial profiles. The remaining 31 samples were swabs taken from various objects including plastic water or soda bottles, glass beer bottles, shot glasses, water or wine glasses, cigarette butts, plastic tip cigar butt, drinking straw, and used condoms. Of these, 71% generated informative profiles, with 23% full profiles, 42% partial profiles, and 6% mixed profiles.

**Table 1:** Rapid DNA results from samples obtained in Costa Rica and Nepal investigations

Sample type	Full profile	Partial profile	Mixture	No DNA	Total No. of samples
Known buccal	18	1	0	0	19
Plastic bottle	1	3	0	0	4
Glass bottle or drinking glass	2	4	0	3	9
Cigarette butt	2	3	0	4	9
Plastic tip cigar butt	0	0	0	1	1
Condom – exterior	0	1	1	1	3
Condom – interior	2	0	0	0	2
Drinking straw	0	2	1	0	3

## The experiences from the TIP investigations

Our experience was based on the investigation of TIP cases in Nepal and Costa Rica. These exploratory field tests included the evaluation of reference samples from victims and evidentiary samples including cigarette butts, water bottles, drinking glasses, and condoms. All of the buccal swabs and 71% of the evidentiary samples provided useful STR profiles, and it is known that not all evidentiary samples will contain DNA. It is apparent that Rapid DNA methods are extremely valuable and can work in challenging environments. Rapid DNA Analysis is well suited for time sensitive field operations and can supplement (but not replace) conventional laboratory methods.

Collection of known buccal samples from TIP victims generated a full STR profile following Rapid DNA analysis 95% of the time. There was no discernible difference in success rates if the samples were collected by trained personnel or by the victims themselves after a very brief description of the process. On several occasions it was necessary for the investigator to demonstrate a buccal swab collection before the victim felt comfortable doing the collection herself.

The majority of items collected from the field and subsequently swabbed for DNA generated partial or full DNA profiles. All drinking containers; plastic water and soda bottles, glass shot glasses, wine glasses, and drinking glasses, as well as straws were able to yield full or partial profiles. Swabs generated from cigarette butts occasionally yielded useful profiles. Profiles were obtained from swabs of the interior surface of the used condom (note that no seminal fluid was observed). Swabs of the exterior surface of the condoms gave results ranging from partial profiles, mixtures, and no profiles.

For samples containing mixtures of seminal fluid and epithelial cells of the victim, it is clear that conventional DNA analysis methods will be superior to Rapid DNA methods. For example, conventional cell separation methodologies and Y-STR analyses are likely to generate more useful information than a simple autosomal STR assay of mixed DNA. In addition, at this point in time samples with low DNA template will likely yield better results with conventional laboratory methods. However, it is very possible that continuing research and improvement with the evolving Rapid DNA technology will result in an increased ability to analyze samples with only trace amounts of DNA.

Currently available DNA swab units were found to be suitable for investigative personnel who need to conceal these items when entering and exiting TIP establishments. The overall size of the individual unit was small enough to avoid detection. Once it was determined that a tube with a drying agent was needed, the overall length of the tube increased. If possible, it would be ideal to minimize the overall size of the collection tube and still maintain the presence of a drying agent. It should be again emphasized that collection of these types of samples in conjunction with ongoing investigations should only be done by properly trained personnel, and in accordance with laws of the country in which the work is being performed. Ideally, these specialized investigators are working in conjunction with and alongside local law enforcement personnel. This approach increases the potential for a long term, sustainable adoption of these methodologies when used to combat human trafficking.

## Conclusions

Overall, it was established that DNA samples obtained by investigators in the course of their ongoing efforts involving cases of sexual exploitation with TIP victims can yield valuable data using Rapid DNA technology. This is extremely promising as Rapid DNA Analysis possesses several key advantages that are beneficial to TIP investigations. The speed at which the results can be obtained is critical; Rapid DNA Analysis can be performed near the site of sample collection, minimizing the time required to process samples. Given the nature of these crimes, and specifically in instances of perpetrators who are engaged in sex tourism, speed is of the essence. With conventional laboratory analysis, perpetrators will have fled the jurisdiction before test results are available; prosecution then becomes increasingly complicated by jurisdiction and extradition issues. Furthermore, personnel with minimal training can perform Rapid DNA Analysis, and there is no need for expensive laboratory facility space. Taken together, these advantages and the work presented here represent an important first step toward the application of Rapid DNA in TIP investigations.

The scope and magnitude of TIP is so much larger than we know or want to acknowledge. While there are many human rights issues which we as a developing, civilized world must address, it is hard to imagine that the enslavement and trauma associated with loss of liberty and sexual exploitation will not rise to the top of that list. While many great efforts to combat TIP have been documented and are still being employed, it is clear that the power of DNA, a time tested effective crime fighting tool, would be of extreme benefit to those who engage in combating this continually-growing criminal enterprise.
